# Using Healthcare Redesign to Identify Medication Management Issues in Parkinson’s Disease

**DOI:** 10.3390/pharmacy13010013

**Published:** 2025-01-30

**Authors:** Susan Williams, Marissa A. Iannuzzi, Sarah J. Prior

**Affiliations:** 1 New South Wales Health, Royal North Shore Hospital, St Leonards, NSW 2065, Australia; susan.williams3@health.nsw.gov.au (S.W.); marissa.iannuzzi@health.nsw.gov.au (M.A.I.); 2 Tasmanian School of Medicine, University of Tasmania, Burnie, TAS 7032, Australia

**Keywords:** Parkinson’s disease, Parkinson disease, medication, medication errors, healthcare redesign, quality and safety, patient experience

## Abstract

Background: Parkinson’s disease (PD) is a neurodegenerative disorder that is predominantly controlled through pharmacotherapy. People with PD have highly complex medication regimens that are often poorly managed during hospital admissions. This project aims to understand the issues experienced by patients with PD and healthcare staff that impacted their medication management during their hospital admission at a tertiary metropolitan hospital in New South Wales, Australia. Methods: This project focuses on the mixed-methods diagnostics phase of the healthcare redesign approach to health service improvement, utilising organisational data, online surveys, interviews, and focus groups. Results: The findings from this project highlight key areas to address to improve the medication management of patients with PD admitted to hospital. The organisational data (*n* = 222) showed that the identification of PD patients, untimely medication reviews, prescribing errors, and untimely medication administration all contributed to poor patient experience. The staff surveys (*n* = 81) highlighted that a lack of knowledge of PD medications and poor patient identification impacted patient experience. The patient surveys (*n* = 18) and patient interviews (*n* = 16) suggested that confidence around medication management and administration timing could be improved. Conclusions: Poor PD medication management in hospital impacts the patient experience and should be improved to ensure better outcomes for patients and the health services.

## 1. Introduction

Parkinson’s disease (PD) is a complex neurodegenerative disorder [1]. The motor symptoms of PD include tremor, rigidity, and postural instability, and the non-motor symptoms include autonomic dysfunction, mood disturbances, cognitive impairment, sleep disorders, and pain [2]. When compared with patients of the same age and gender, patients with PD admitted to acute care hospitals in Australia are five times more likely to be treated for delirium, three times more likely to experience adverse drug events and syncope, and more than twice as likely to require management for falls with injury, dementia, gastrointestinal complications, urinary tract infections, and reduced mobility [3]. Hospital admissions are associated with worse outcomes, often resulting in the worsening of motor symptoms, medication errors, hospital-acquired complications, longer lengths of stay, and higher readmission rates [4]. The early identification of this vulnerable cohort of patients enables clinicians to recognise those at risk of complications early and work with multidisciplinary teams to reduce the impact of these complications on the patient and their hospital stay [3].

People with PD often have highly individualised, complex medication regimens. Frequently, these regimens are incorrectly prescribed and administered in hospital [5]. A partnership approach between patients with PD and the healthcare team is required for the delivery of medications to the patients at the correct, individualised times. Medical officers are required to prescribe medication appropriately and accurately, and nursing staff are required to administer the prescribed medications in accordance with the given orders [6,7]. However, the timely administration of PD medications, particularly those containing levodopa, in hospital is poor [6]. The timely administration of levodopa has been shown to be difficult due to the limited awareness of staff regarding the importance of the timely administration for PD symptom control and the limited availability of the medication [8]. Globally and in Australia, only half of PD medication doses are given in a timely manner in hospital [1,9]. The importance of improving the prescribing accuracy and timely administration of PD medications in hospital is clear when considering the consequences of medication mismanagement. Non-adherence to PD patients’ individual medication regimens in hospital contributes to the fact that patients with PD are 1.5 times more likely than patients without PD to have a longer length of stay [6]. Untimely administration can worsen symptoms, which can cause hospital-acquired complications, such as delirium, falls, and constipation, placing a burden on the healthcare system [6]. Although the prescribing accuracy of PD medication management and the administration in hospital is multifactorial, pharmacists play an integral role in this process. Proactive interventions utilising pharmacy technology, workflow, and staffing have previously been shown to improve accuracy and safe hospitalisation for people with PD [10]. Unless the identification of PD patients occurs earlier and their medications are managed efficiently and effectively, PD patients’ symptoms will continue to worsen in hospital. This will consequently worsen their hospital experience and health outcomes and increase their length of stay [3,4,6]. Harris et al. [11] demonstrated that the clear and early identification of PD patients within the emergency department (ED) reduced their length of stay in the ED. The effective identification of PD patients improved the timeliness of PD medication administration, the prescribing accuracy of PD medications, and the timeliness of a medication reconciliation review by a pharmacist. Medication reconciliation—the identification of current medications and the regime in which they are taken— is an important factor in the safe care of patients with PD. Previous studies have suggested that medication reconciliation has a positive impact on reducing the length of stay and mortality rate by reducing medication errors by 51% [12].

Healthcare redesign is an approach to health service improvement that involves understanding and investigating the root causes of an identified health systems and processes problem. Developing evidence-based, co-designed solutions for meaningful and sustainable outcomes is integral to healthcare redesign, which makes this approach suitable for medication management issues in acute care. Previous studies using this approach have demonstrated positive outcomes for PD patients [13]. Healthcare redesign comprises five main phases for successful health service improvement. Firstly, the scoping phase involves the identification of a healthcare service delivery problem and the development of a project aim to improve the current situation. The aim should consider three viewpoints: patients, healthcare workers, and the healthcare organisation [14]. Furthermore, defining the scope of the problem and understanding the sphere of influence is critical in the scoping phase of redesign. The second phase of redesign, diagnostics, involves understanding the current, baseline situation and how it contributes to the identified problem. The following three phases, solution design, implementation, and evaluation, focus on developing evidence-based, co-designed solutions, and the implementation and evaluation of those solutions.

Limited literature exists on the underlying causes of suboptimal PD medication management in hospital. Therefore, the aim of this project was to identify and understand (diagnose) the issues experienced by PD patients and healthcare staff that impacted their medication management during hospital admission at a tertiary metropolitan hospital in New South Wales, Australia.

## 2. Materials and Methods

### 2.1. Setting

Royal North Shore Hospital (RNSH) is the principle tertiary referral hospital for Northern Sydney Local Health District (NSLHD) with a maximum capacity of 420 acute medical and surgical beds. This project focusses on PD patients across three wards—ED, Neurology, and Aged Care. The project period for this work was from 1 January 2022 to 31 May 2022.

The hospital uses an electronic medical record (eMR) system that includes electronic documentation, medication prescribing, and administration (Epic Systems, Wisconsin, USA). In the ED, as part of the eMR, the FirstNet program, a system for managing admissions, vacant beds, patient waiting times, and the availability of medical test results, also includes the ability to triage and tracking screens displaying real-time information for the ED. Patients with the FirstNet problem code ‘Parkinson’s disease 81717011′ entered into eMR are identified in the ED with a green PD Icon on the FirstNet tracking visual display board upon arrival to the ED.

### 2.2. Procedure

**Figure 1:**
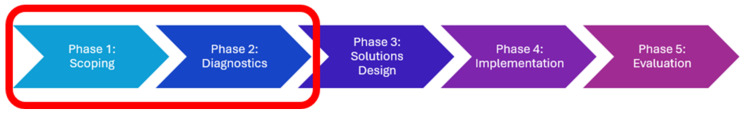
Five phases of Healthcare Redesign.

This project utilised the principles of the healthcare redesign methodology [14] (Figure 1) and explored Phase 1 (Scoping) and Phase 2 (Diagnostics).

A mixed-methods approach was utilised in the diagnostics phase of this redesign project to understand the problem of poor PD medication management in hospital. Table 1 describes the methods used.

Ethics approval for this investigation was provided by the research Office of Western Sydney Local Health District Research and Education Network, 2019/ETH10758. This project was part of the University of Tasmania, Graduate Certificate (Clinical Redesign) program.

### 2.3. Data Analysis

Quantitative data were analysed with descriptive statistics using Microsoft Excel (Version 2410). This included percentages to summarise the characteristics of our numerical data. Qualitative data from the interviews and the focus group session were transcribed, coded, and themed using Microsoft Excel. A general inductive approach was utilised to analyse interview and focus group data [15]. A process map was developed to show steps in the patient journey and issues identified at each step or process. A prioritisation matrix [16] was utilised to prioritise the findings for further investigation, and root cause analysis (5 whys) [17] was conducted to understand the prioritised issues. The project steering committee rated the impact and priority of the root causes analysis.

## 3. Results

A total of 222 PD patients were admitted to RNSH during the project period. Of these 222 patients, 179 were admitted via the ED, 33 to the Neurology ward, and 31 to the Aged Care ward. Eight of the patients admitted to Neurology were booked admissions and did not pass through the ED, and only one patient was a booked admission to Aged Care. A total of 158 patients were admitted to other wards outside the scope of this project.

PD was the primary reason for admission in 7% (n = 20) of the PD patients. PD was considered an ‘inactive’ diagnosis in 61% (n = 135) of the hospital admissions. The median length of stay (LOS) was 11 days across the whole hospital. The average PD patient was 79 years old, and 60% were male (n = 133) (Table 2).

### 3.1. Organisational Data

The organisational data show that there were four main areas impacting patient experience (Table 3). These are discussed below.

#### 3.1.1. Identification of PD Patients

A total of 39% (*n* = 69) of PD patients admitted via the ED could be identified using the green icon alert on the FirstNet board.

#### 3.1.2. Untimely Pharmacist Medication Review

A total of 27% (*n* = 59) of all admissions received a pharmacist medication reconciliation prior to discharge. A total of 29% (*n* = 9) and 45% (*n* = 14) of PD patients received a pharmacist medication reconciliation prior to discharge on the Neurology and Aged Care wards, respectively. Overall, the proportion of PD patients with a pharmacist medication reconciliation within 48 h of admission was 16% (*n* = 35).

#### 3.1.3. Prescribing Errors

A total of 77% (*n* = 33) of pharmacist medication reconciliation (*n* = 50) documented at least one PD prescribing error, with 32% of errors related to incorrectly prescribed times. Other error types included incorrect dose (20%), incorrect formulation (12%), and incorrect medication (9%).

#### 3.1.4. Untimely Administration

Hospital-wide, the organisational data show that 52% of levodopa doses were given on time, within 15 min of the prescribed time. Totals of 33%, 62%, and 46% of levodopa doses were administered on time in the ED, Neurology ward, and Aged Care wards, respectively.

### 3.2. Surveys

#### 3.2.1. Staff Surveys

A total of 81 staff completed the survey (Table 4).

A total of 63% (*n* = 51) of staff did not know that the administration of PD medications ‘on time’ means within 15 min of the prescribed time. Only 13% of medical officers were confident that the PD medications were prescribed accurately. Totals of 68% of medical officers, 69% of nurses, and 38% of pharmacists were satisfied with the level of care that they were able to provide people living with PD.

A total of 14% (*n* = 21) of pharmacists agreed that people with PD were readily identifiable to them. A total of 58% of other health staff (*n* = 47) agreed that PD patients were readily identifiable to them. A total of 64% (*n* = 52) of all hospital staff agreed they were always aware when a patient under their care had PD, while 67% of pharmacists disagreed.

#### 3.2.2. Patient Surveys

A total of 18 patients and 14 carers completed the survey (*n* = 32). A total of 40% (*n* = 6) of patients were unsure which ward they had been admitted to. The patients and carers reported that their medications were not given on time. A total of 81% of overall participants (*n* = 26) reported that 75% or more of their PD medication doses were given on time in the ED, and 69% (*n* = 22) suggested that 75% or more of their PD medication doses were given on time on the Neurology ward and Aged Care ward.

### 3.3. Qualitative Data

#### 3.3.1. Patient Interviews

Interviews were conducted with 16 patients and carers. The patients and their carers perceived that their medications were not provided on time during their hospital admission.

*“My medications were not given on time”*

—Patient 2.

*“The medications were later than we would have liked”*

—Patient 5.

#### 3.3.2. Focus Groups—Process Mapping

The focus group session was attended by a range of multidisciplinary health professionals (Table 5).

**Figure 2:**
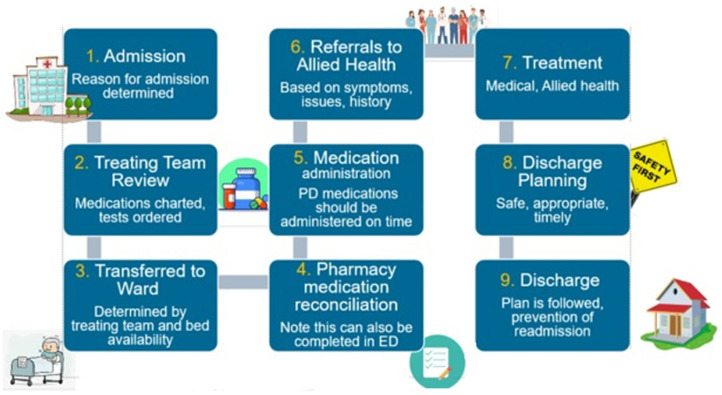
Process map of typical PD patient journey.

A process map was developed based on the journey of a typical patient, from the perspective of healthcare professionals who participated in the focus group sessions (Figure 2).

A total of 160 lines of data were collected, with 93 lines related to the management of PD medications. The discussion focussed on general issues around medication management, with some specific feedback around areas of concern.

*“People do not pay attention to the specifics of the timing of medications”*

—Staff 11.

*“…There are delays in getting medications from pharmacy in a timely fashion”*

—Staff 4.

*“Doctors not good at the timing of medications”*

—Staff 18.

*“PD medication literacy problem in general”*

—Staff 28.

General inductive thematic analysis identified 11 categories of issues.

1. Pharmacist reviews of PD patients are not conducted in a timely fashion.
2. PD medications are incorrectly prescribed.
3. PD medications are often not administered on time.
4. Staff lack knowledge about PD medications.
5. Medications are not charted in a timely fashion.
6. PD patients are not readily identifiable to hospital staff.
7. There is a lack of clarity and structure in addressing PD patients’ care needs.
8. Patients do not receive a comprehensive PD specific nursing care assessment or coordination of care.
9. Staff lack knowledge about PD management.
10. There is poor continuity and coordination of care upon discharge.
11. Swallow safety assessment does not always occur in a timely fashion.

The prioritisation matrix was then used to determine which issues to address first. The most important issues were considered to have the highest impact on hospital key performance indicators and the highest feasibility to implement. Six issues were selected for prioritisation (Table 6).

Root cause analysis was conducted for each of the six issues. Table 6 shows that the three issues with the highest impact and highest priority are ‘PD patients are not readily identifiable to hospital staff’, ‘Pharmacist reviews are not conducted in a timely fashion’, and ‘Staff lack knowledge about PD medications’. The root causes identified for these issues are related to staff knowledge and awareness, competing priorities, and identification issues.

## 4. Discussion

This healthcare redesign project aimed to explore and understand the medication management issues that affect PD patients in hospital. The most significant factors contributing to poor medication management during hospital admission were poor patient identification, untimely pharmacist reviews, medication prescribing errors, untimely medication administration, poor staff knowledge of PD medications, and PD medications not charted in a timely manner.

It is estimated that 0.8% of the urban population around the RNSH live with PD [7], which equates to approximately 7885 people living with PD in the local health district. Previous work with RNSH organisational data (2021) demonstrated that only half of PD medication doses were administered on time, which is consistent with the literature [1,6,11]. Furthermore, 45% of pharmacist reviews at RNSH identified a PD prescribing error, and there was no systematic means of identifying PD patients. The identification of PD patients in an acute care hospital has been shown to improve outcomes, such as the accurate prescribing of medication, improved timeliness of medication administration, and increased pharmacist medication reconciliation [13,18,19,20]. While a system for the identification of PD patients currently exists within the ED at RNSH, this project identified that many staff were not aware of it, and it is not routinely used. Additionally, this system of identification is not integrated beyond the ED, meaning that there is currently no specific method of identifying PD patients on the Neurology ward or the Aged Care ward. Without clear identification of PD patients in the ED and on the wards, pharmacists, doctors, and nurses are unable to effectively prioritise reviewing PD medications, prescribing PD medications, and administering PD medications. Mistakes are easily made when prescribing PD medication regimens, making pharmacist reviews important, as prescribing errors are more likely to be identified. If such errors are not detected efficiently, they are often perpetuated throughout the admission. This can result in poor PD symptom control and poor outcomes for patients.

PD patients will frequently come into contact with medical staff who have limited expertise in PD, which creates significant challenges throughout their hospital admission [1]. Medical officers at RNSH were not confident that medications were prescribed accurately. Medication timing errors were the most common error found. PD medications were often not administered on time, partly because the nursing staff were not aware that ‘on time’ means within 15 min of the prescribed time, according to the NSW Health safety notice [7]. Access to PD medications and an eMR system that does not advocate for timely administration and workload also contributed to untimely medication administration, which is consistent with a previous study looking at pharmacy strategies for improving medication administration [10]. The staff indicated that their knowledge of PD medications was poor, and there was limited routine education provided to specifically address the knowledge gap for accurate prescription, efficient review, and timely administration. Multiple studies have shown that this knowledge gap is common, and improvements to medication management can be achieved with the implementation of education programs within acute care hospitals [6,8,21,22,23,24]. Interestingly, despite the hospital’s poor performance, the patients and carers perceived that their doses were administered on time.

The next phase of this healthcare redesign project is the development of solutions based on the findings from the current phase. The project team will engage with all stakeholders involved in the diagnostics phase of PIE1 and co-design solutions to meet the aims of the overarching project to improve the health outcomes, experience of care, and service efficiency though excellent inpatient medication management for PD patients admitted to the Emergency Department, Neurology, and Aged Care wards at Royal North Shore Hospital, Sydney, Australia.

### Limitations

This study was conducted in three specific areas; therefore, the findings are not necessarily generalisable to other hospital locations. However, we can hypothesise that other locations may demonstrate even poorer PD medication management due to less PD expertise. Furthermore, the sample size of the surveys, focus groups, and interviews may limit the robustness of the findings. The measurement of prescribing errors in this project was pharmacist documentation. There are likely to be more prescribing errors which were not identified in this project, because pharmacists are unable to review all PD patients. Additionally, some pharmacists may have identified errors but may have verbally communicated these to the medical team rather than document them. This project only focused on the proportion of levodopa doses administered on time and did not include other medicines indicated for PD.

## 5. Conclusions and Prospects

The healthcare redesign methodology, diagnostic phase has successfully identified the medication management issues through the experiences of staff and patients or carers and organisational data. The poor identification of PD patients, untimely medication review, prescribing errors, and the untimely administration of levodopa medications contributed to a poor experience for PD patients admitted to RNSH. This information will be utilised to inform evidence-based solutions to improve the care and service delivery for PD patients at RNSH.

## Figures and Tables

**Table 1:** Methods utilised in the diagnostic phase.

Diagnostic Tool	Purpose	Source
Organisational data	To identify the number of PD patients admitted to RNSH through the ED to the Neurology and Aged Care wards.	RNSH admissions data from 1 January 2022–31 May 2022.
	To determine the following: The proportion of pharmacist medication reconciliation review notes in the electronic medical record (eMR) which reported a PD medication prescribing error. The proportion of pharmacist medication reconciliation reviews which were documented within 48 h of admission and prior to discharge. The proportion of levodopa doses administered on time (defined as 15 min within the prescribed time [8]). The proportion of patients correctly identified in the ED as having PD, with a ‘PD icon’ in the eMR.	Automated reports: PD icon usage (ED) to identify PD patients upon arrival. Pharmacy medication reconciliation conducted. Administration of medications containing levodopa. Manual data extraction from pharmacy medication reconciliations.
Online surveys	To understand how healthcare staff identified PD patients in hospital under their care, and their knowledge of the importance of PD medication timing and accuracy. The survey was piloted with 10 staff members prior to going live.	Electronic survey—9-question Likert scale.
	To understand the patient and carer perspective on staff performance in managing their PD medications during their hospital admission. The survey was piloted with 11 patients and 9 carers before going live.	Electronic survey—10-Question Likert scale.
Interviews	To understand patient and carer experiences of PD management in hospital.	Patients and carers. Telephone interviews transcribed into Excel spreadsheet.
Focus groups	To map out workflow processes, identify issues, and gain perspective on caring for PD patients in collaboration with staff.	Nurses. Allied Health professionals. Pharmacists. Geriatricians Neurologists. Audio recorded and transcribed into Excel spreadsheet.
Literature review	Understand the current literature	Database search.

**Table 2:** Demographic data of admitted PD patients at RNSH from January to May 2022.

	RNSH	Neurology	Aged Care	Emergency
	(n = 222)	(n = 33)	(n = 31)	(n = 179)
Male,	133 (60%)	16 (48%)	20 (65%)	98 (55%)
Age, median (IQR)	81 (74–85)	78 (71.25–84)	85 (82.5–89.5)	81.5 (75–86)
Number of admissions with PD as the reason for admission	20 (9%)	13 (39%)	4 (13%)	17 (9%)
Number of admissions with ‘active’ diagnosis of PD-ICD10 code G20	87 (39%)	23 (70%)	17 (55%)	76 (42%)
Number of admissions with an ‘inactive’ diagnosis of PD-ICD10 code U80.1	135 (61%)	10 (30%)	14 (45%)	103 (58%)
Number of planned admissions	43 (19%)	8 (24%)	1 (3%)	Not applicable

**Table 3:** Organisational data.

	RNSH	Neurology	Aged Care	ED
	(n = 222)	(n = 33)	(n = 31)	(n = 179)
Admissions identified in the ED by the green PD icon on the FirstNet board	-	-	-	39 (*n* = 69)
Admissions with a pharmacist medication reconciliation prior to discharge, %	(*n* = 59)	(*n* = 9)	(*n* = 14)	(*n* = 52)
	27	29	45	30
Admissions with a pharmacist medication reconciliation within 48 h of admission, %	(*n* = 35)	-	-	-
	16			
Medication reconciliations with prescribing errors, %	(*n* = 17)	-	-	-
	33			
Number of levodopa doses administered	3699	1396	808	352
Levodopa doses administered on time (within 15 min of the prescribed time), %	(*n* = 3309)	(*n* = 860)	(*n* = 374)	(*n* = 117)
	52 (1.5%)	62 (7.2%)	46 (12.3%)	33 (28%)

**Table 4:** Demographic data of healthcare staff survey participants.

Role	Number of Staff Participants (*n* = 81)
Pharmacist	21 (26%)
Nurse	35 (43%)
Medical Officer	25 (31%)

**Table 5:** Demographics of process mapping focus group participants.

Role	Number at Focus Group
Emergency Department Nurse	10
Neurology Nurse or Allied Health	15
Aged Care	18
Pharmacist	31
Geriatrician	8
Neurologist	5

**Table 6:** Root cause analysis results.

Key Issue	Root Cause	Impact	Priority
PD patients are not readily identifiable to hospital staff.	Staff are unaware that the PD alert icon in FirstNet exists and what it achieves	High	High
	Staff are unaware of how to set up the PD alert icon in FirstNet	High	High
	No alert system for the identification of PD patients on the ward	High	High
	No bedside (non-electronic) means of identification	High	High
2. Pharmacist reviews of PD patients are not conducted in a timely fashion.	Insufficient funding to increase pharmacist-to-patient ratios	High	Medium
	Pharmacists have competing priorities	High	High
	PD patients are not readily identifiable to pharmacists in the ED due to poor use of the electronic PD alert icon	High	High
	No alert system exists in the eMR for the identification of PD patients on wards	High	High
3. Staff lack knowledge about PD medications.	Lack of education on PD medications	High	High
4. PD medications are often not administered on time.	Competing nursing priorities prevent timely administration from being prioritised	High	Low
	Lack of education regarding the importance of administering medications on time	High	High
	eMR system does not advocate for timely administration of PD medications	High	High
	High-risk or low-use medications may not be available on the ward	Medium	Medium
	Delays in obtaining medication which is not available on ward—there is no alert system for pharmacists and nurses to order medications available only in the dispensary	Medium	Medium
5. PD medications are often incorrectly prescribed.	Medication regimens are complex and the history is poorly taken due to heavy workloads and a lack of education	High	Medium
	The eMR function to chart custom medication administration times is not user-friendly	High	High
	There is a lack of education on how to chart custom medication administration times	High	High
6. PD medications are not charted in a timely fashion.	Doctors often do not prioritise charting complex medication regimes until the admission is confirmed	Medium	Medium
	Due to the complexity of the medication regimens, doctors often prefer to wait for a pharmacist review, which delays charting	Low	Low
	Other urgent tasks are often prioritised above the charting of PD medications (which may often be appropriate)	Low	Low
	Doctors’ high workload	Medium	Low
	Junior doctors may need to liaise with senior doctors if medication changes are necessary, for example, in patients with swallowing difficulties, which can delay charting	Low	Low

## Data Availability

The data presented in this study are available upon request from the corresponding author due to ethics approval restrictions. Additional information can be found in the Supplementary Materials.

## Supplementary Materials

The following supporting information can be downloaded at: https://www.mdpi.com/article/10.3390/pharmacy13010013/s1.
