# Supplementary material for: Using Healthcare Redesign to Identify Medication Management Issues in Parkinson’s Disease
Source: Pharmacy (Basel). 2025 Jan 30;13(1):13. doi: 10.3390/pharmacy13010013 (PMC11859038; doi:10.3390/pharmacy13010013)
Supplement: Supplementary file 1 [file pharmacy-13-00013-s001.zip › Survey-Patient.pdf]

PARKINSON INPATIENT EXPERIENCE

# Parkinson Inpatient Experience (PIE) Patient Survey

## Thank you for participating in this survey

**This information page is to tell you about a research study that is being conducted at Royal North Shore Hospital by Elizabeth Bryan (Service Improvement Manager), Sue Williams (Parkinson Clinical Support Nurse) and Marissa Sakiris (Pharmacist). The purpose of this research is to improve the health outcomes and hospital experience for people living with Parkinson Disease and their carers. People with Parkinson Disease often have a longer length of hospital stay and worse health outcomes, compared with people without Parkinson Disease. As part of this research, we are seeking to better understand the hospital experience of people with Parkinson Disease and their carers at Royal North Shore Hospital.**

**We invite you to take part in this research project by completing this survey. The survey should take less than 5 minutes to complete. Participation in this study is voluntary. Although you may not benefit directly from this research, we hope that the study will help capture the hospital experience of Parkinson Disease patients, and improve our service in the future.**

**If you don't wish to take part, please disregard this information and simply close the window of your web browser.**

**The information collected from you in this study will be non-identifiable and will only be used for the purposes of this study. Your survey responses will be kept confidential and only Elizabeth Bryan, Sue Williams and Marissa Sakiris will have access to this data. We plan to present the study results to staff at Royal North Shore Hospital. Results of the survey maybe published as part of a wider project however you will not be identifiable in any publication or presentation.**

**If you complete the survey, we will assume that you have given consent to take part in this study. As your survey responses are non-identifiable, if you complete the survey and then later decide that you don't want to take part, we will not be able to delete your survey responses.**

**If you would like to ask any questions about the study, please contact the researcher Sue Williams at susan.williams3@health.nsw.gov.au, who will be happy to discuss the study with you.**

**This study has been approved by the Western Sydney Local Health District Human Research Ethics Committee (2019/ETH10758) as part of a wider study by the NSW Agency for Clinical Innovation and the University of Tasmania. Any person with concerns or complaints about the conduct of this study should contact the Research Office who is nominated to receive complaints from research participants. You should contact them on phone number 02 8890 9007 and quote the reference number 2019/ETH10758.**

**Thank you for taking the time to consider this study.**

* 1. Are you living with Parkinson Disease AND were you admitted to Royal North Shore Hospital in the last 6 months?

If your answer to this question is yes, please answer the following questions in relation to your time in the **Emergency Department** this hospital admission.

- ◯ Yes
- ◯ No

* 2. How often were your Parkinson Disease medications given on time?
'On time' is considered to be within 15 minutes of the time they are supposed to be taken.
Choose the option that most closely represents your experience.

- ◯ 100% of the time
- ◯ 75% of the time
- ◯ 50% of the time
- ◯ 25% of the time
- ◯ 0% of the time
- ◯ Unsure

* 3. I was satisfied that my medications were managed well by hospital staff.
Good medication management refers to the correct medications being administered in a timely manner.

- ◯ Strongly agree
- ◯ Agree
- ◯ Neither agree nor disagree
- ◯ Disagree
- ◯ Strongly disagree
- ◯ Unsure

* 4. I felt staff were aware that I had Parkinson Disease.

- ◯ Strongly agree
- ◯ Agree
- ◯ Neither agree nor disagree
- ◯ Disagree
- ◯ Strongly disagree
- ◯ Unsure

* 5. Please provide any comments about your **Emergency Department** experience that you would like to share with us.
Please remember to ensure your answer does not identify you specifically.

PARKINSON INPATIENT EXPERIENCE

## Parkinson Inpatient Experience (PIE) Patient Survey

**Please answer the following questions in relation to your time in the ward this hospital admission.**

* 6. What ward were you admitted to?

- ◯ 7F Neurology
- ◯ 9E Aged Care
- ◯ Unsure

* 7. How often were your Parkinson Disease medications given on time?
'On time' is considered to be within 15 minutes of the time they are supposed to be taken.
Choose the option that most closely represents your experience.

- ◯ 100% of the time
- ◯ 75% of the time
- ◯ 50% of the time
- ◯ 25% of the time
- ◯ 0% of the time
- ◯ Unsure

* 8. I was satisfied that my medications were managed well by hospital staff.
Good medication management refers to the correct medications being administered in a timely manner.

- ◯ Strongly agree
- ◯ Agree
- ◯ Neither agree nor disagree
- ◯ Disagree
- ◯ Strongly disagree
- ◯ Unsure

* 9. I felt staff were staff aware that I had Parkinson Disease.

- ◯ Strongly agree
- ◯ Agree
- ◯ Neither agree nor disagree
- ◯ Disagree
- ◯ Strongly disagree
- ◯ Unsure

* 10. Please provide any comments about your **ward** experience that you would like to share with us.
Please remember to ensure your answer does not identify you specifically.

PARKINSON INPATIENT EXPERIENCE

Parkinson Inpatient Experience (PIE) Patient Survey

## Thank you for participating in this survey

**The researches would like to interview 10 people to gain further insight into the hospital experiences of people living with Parkinson Disease. If you are willing to share your story in more detail, please contact Sue Williams Parkinson Disease Nurse susan.williams3@health.nsw.gov.au to register your interest as soon as possible.**
