# Supplementary material for: Using Healthcare Redesign to Identify Medication Management Issues in Parkinson’s Disease
Source: Pharmacy (Basel). 2025 Jan 30;13(1):13. doi: 10.3390/pharmacy13010013 (PMC11859038; doi:10.3390/pharmacy13010013)
Supplement: Supplementary file 1 [file pharmacy-13-00013-s001.zip › Survey-Staff.pdf]

PARKINSON INPATIENT EXPERIENCE

Parkinson Inpatient Experience (PIE) Staff Survey

Thank you for participating in this survey

This information page is to tell you about a research study that is being conducted at Royal North Shore Hospital by Elizabeth Bryan (Service Improvement Manager), Sue Williams (Parkinson Clinical Support Nurse) and Marissa Sakiris (Pharmacist). The purpose of this research is to improve the health outcomes and hospital experience for people living with Parkinson Disease and their carers. People with Parkinson Disease often have a longer length of hospital stay and worse health outcomes, compared with people without Parkinson Disease. As part of this research, we are seeking to better understand the experience of staff at Royal North Shore Hospital in caring for patients with Parkinson Disease.

We invite you to take part in this research project by completing this survey. The survey should take less than 5 minutes to complete. Participation in this study is voluntary. Although you may not benefit directly from this research, we hope that the study will help capture the experience of hospital staff caring for patients with Parkinson Disease and improve our service in the future.

If you don't wish to take part, please disregard this information and simply close the window of your web browser.

The information collected from you in this study will be non-identifiable and will only be used for the purposes of this study. Your survey responses will be kept confidential and only Elizabeth Bryan, Sue Williams and Marissa Sakiris will have access to this data. We plan to present the study results to staff at Royal North Shore Hospital. Results of the survey maybe published as part of a wider project however you will not be identifiable in any publication or presentation.

If you complete the survey, we will assume that you have given consent to take part in this study. As your survey responses are non-identifiable, if you complete the survey and then later decide that you don't want to take part, we will not be able to delete your survey responses.

If you would like to ask any questions about the study, please contact the researcher Sue Williams at susan.williams3@health.nsw.gov.au, who will be happy to discuss the study with you.

This study has been approved by the Western Sydney Local Health District Human Research Ethics Committee (2019/ETH10758) as part of a wider study by the NSW Agency for Clinical Innovation and the University of Tasmania. Any person with concerns or complaints about the conduct of this study should contact the Research Office who is nominated to receive complaints from research participants. You should contact them on phone number 02 8890 9007 and quote the reference number 2019/ETH10758.

Thank you for taking the time to consider this study.

* 1. In what area of the hospital do you work?

- Emergency Department
- Neurology 7F
- Aged Care 9E
- My position rotates regularly

* 2. I am satisfied with the level of care I am able to provide people living with Parkinson Disease.

- Strongly agree
- Agree
- Neither agree nor disagree
- Disagree
- Strongly disagree

* 3. People with Parkinson Disease are readily identifiable within the hospital to me.

- Strongly agree
- Agree
- Neither agree nor disagree
- Disagree
- Strongly disagree

* 4. I am always aware when a patient under my care has Parkinson Disease.

- Strongly agree
- Agree
- Neither agree nor disagree
- Disagree
- Strongly disagree

* 5. How do you identify that a patient under your care has Parkinson Disease?
You may choose more than 1 answer.

- [ ] Powerchart - Census Task List
- [ ] Written handover
- [ ] Verbal handover
- [ ] FirstNet Enhanced Tracking Board - listed in presenting problem
- [ ] FirstNet Enhanced Tracking Board - '**PD**' icon
- [ ] Board round
- [ ] Powerchart documentation
- [ ] Other (please specify)

* 6. A __________minute delay in Parkinson Disease medication dosing may make a significant difference to symptom control.

- 5
- 10
- 15
- 30
- 45

* 7. The timing of Parkinson Disease medications is always important to my role.

- Strongly agree
- Agree
- Neither agree nor disagree
- Disagree
- Strongly disagree

* 8. I would like further training in the management of patients with Parkinson Disease.

- Yes
- No

* 9. What is your role?

- Nurse
- Doctor
- Pharmacist
- Physiotherapist
- Other (please specify)
- Occupational Therapist
- Dietitian
- Social Worker
- Speech Pathologist

PARKINSON INPATIENT EXPERIENCE

Parkinson Inpatient Experience (PIE) Staff Survey

* 10. I know how to prescribe a medication to be given four times a day at specifically 7am, 11am, 3pm, 7pm.

- Yes
- No - I would need to ask someone for guidance.

* 11. I am always confident that Parkinson Disease medications prescribed on admission are consistent with the medication regime taken pre-admission, including correct medication names, administration times and dosage forms.

- Strongly agree
- Agree
- Neither agree nor disagree
- Disagree
- Strongly disagree

* 12. What are three common medications which are contraindicated in people with Parkinson Disease?

PiE
PARKINSON INPATIENT EXPERIENCE

Parkinson Inpatient Experience (PIE) Staff Survey

* 13. What are three common medications which are contraindicated in people with Parkinson Disease?

PiE
PARKINSON INPATIENT EXPERIENCE

Parkinson Inpatient Experience (PIE) Staff Survey

Thank you for participating in this survey.

* 14. Please provide any comments or ideas you would like to share with us.
Please remember to ensure your answer does not identify you specifically.
